# Lung Cancer Immunotherapy Approaches: From Clinical Testing to Future Advances

**DOI:** 10.34172/apb.025.45104

**Published:** 2025-06-02

**Authors:** Mayuri Bhattacharyya, Suktilang Majaw

**Affiliations:** Department of Biotechnology and Bioinformatics, North-Eastern Hill University, Shillong, India 793022

**Keywords:** Adoptive cell therapy, Clinical trial, Immune, Checkpoint inhibitors, Immunotherapy, Lung cancer, Vaccines

## Abstract

One of the major reason of deaths due to cancer globally is caused by lung cancer of which the two main types include non-small cell and small cell lung cancer. The onset of treatment-resistance in cancer cells offers a serious obstacle to the therapeutic effect despite that primary conventional treatments have provided significant benefits and cures. Cancer immunotherapy offers a compelling alternative in patients by utilizing their immune system to enhance its ability to fight against tumors. Cancer immunotherapy includes treatment with cytokines, hormones, bacterial products, monoclonal antibodies, vaccines, etc. Many of these immunotherapies are clinically tested in lung cancer patients. Tumor-associated antigens specific for lung cancer are being targeted using monoclonal antibodies and vaccines. Genetically engineered T-cells that are cultured outside the body are reinfused into the patients. In this review, we describe the different immunotherapeutic approaches that have been clinically tested and used to treat lung cancer globally. The data presented are collected from published studies through electronic databases like Google Scholar and Pubmed using keywords like immunotherapy, adoptive cell therapy, cancer, vaccines, lung cancer and immunological checkpoint inhibitors. The clinical trial results were acquired from ClinicalTrials.gov.in, a database of clinical research studies and their result updates. The review examines the current immunotherapies available for lung cancer treatment globally. While these therapies offer significant benefits to the community, several challenges have hindered their widespread adoption. Key issues such as adverse effects, high costs and varying patient responses to lung cancer immunotherapy require careful consideration. The integration of advanced technologies, including artificial intelligence and bioinformatics tools, along with combinatorial strategies and thorough monitoring, has the potential to increase widely use of lung cancer immunotherapy.

## Introduction

 Lung cancer makes up 11.4 percent of all cancer cases and is responsible for 18 percent of all cancer-related deaths, has transformed over the last century from a rare and little-known illness into the most prevalent cancer worldwide. This shift has established it as a leading factor to cancer-related morbidity.^1,2^ Histologically lung cancer is categorized as small cell lung cancer (SCLC), accounting for 15 percent and non-small cell lung cancer (NSCLC), accounting for 85 percent of the total lung cancer patients.^3-6^ For patients in the initial stages of NSCLC, a surgical intervention is the preferred method, but even in cases of complete tumor removal; there is a substantial chance of cancer recurrence and associated morbidity. It is advisable to use post-operative platinum-derived chemotherapy to lower the likelihood of the disease recurrence rate.^7^ Chemotherapy can extend overall survival (OS) and progression free survival (PFS) in patients of late-stage NSCLC by up to 12-18 and 4-6 months, respectively.^8,9^ In the past ten years, several drug targeting strategies have developed, but drug resistance and inefficiency remain a significant hindrance in cancer treatment.^10^ One alternative method of cancer treatment is utilizing the immune system to stimulate a response targeting cancer. Immunotherapy offers a more precise and effective alternative to traditional treatments such as radiation and chemotherapy typically impact healthy and cancer cells indiscriminately. The innovations in immunotherapy represent cutting-edge technologies to improve cancer treatment and overcome drug resistance in cancer cells.^11^

**Figure 1:**
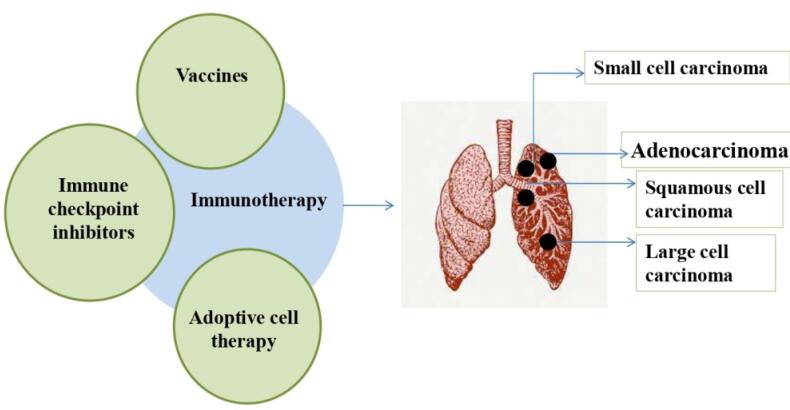
Immunotherapies for lung cancer treatment: The immunotherapies that are currently available to treat various lung cancer kinds are depicted in the figure (Left). The figure (right) shows the lung cancer types-adenocarcinoma, squamous cell carcinoma, large cell and small cell carcinoma. ICIs, vaccines, and adoptive cell therapy are the immunotherapies that have been used for treating lung cancer

 The first cancer immunotherapy was Coley’s toxin, a concoction of live but dormant strains of *Serratia marcescens* and *Streptococcus pyogenes*. However, the absence of understanding of the underlying mechanism and the probable risk factors associated with its induction into cancer patients was not encouraged. The lack of relevant information and research on cancer immunotherapy caused oncologists to proceed with traditional therapeutic approaches like chemotherapy, radiotherapy, and surgery in the 20^th^ century. With the advancement of new technologies in the 20^th^ century, Paul Ehrlich, F. Macfarlane Burnet, and Lewis Thomas individually hypothesized the concept of ‘immune surveillance’. As per the ‘cancer immune surveillance’ hypothesis, immune system identifies and attacks neoantigens associated with tumors preventing carcinogenesis. However, the host immune response can play a dual role of destroying and shaping the cancer cells through different phases of cancer immunoediting.^12,13^ During the immunoediting process, one of the escape mechanisms used by tumor is via formation of an immunosuppressed tumor microenvironment (TME) by cytokines secretion and overexpression of co-inhibitory immune checkpoints.^14^ Targeted medicines, such as immunotherapy, have been developed for advanced cancer treatment by targeting the molecular pathways involved in cancer immune evasion. Here, we have evaluated the clinically tested immunotherapies that have been used in the lung cancer treatment. This review summarizes different types of immunotherapies and their clinical trial outcomes, including the approved immunotherapies and novel targets to treat lung cancer. The immunotherapies currently being tested and used for lung cancer treatment are indicated in Figure 1; the details of which are given in the next section.

## Immune checkpoint inhibitors (ICIs)

**Figure 2:**
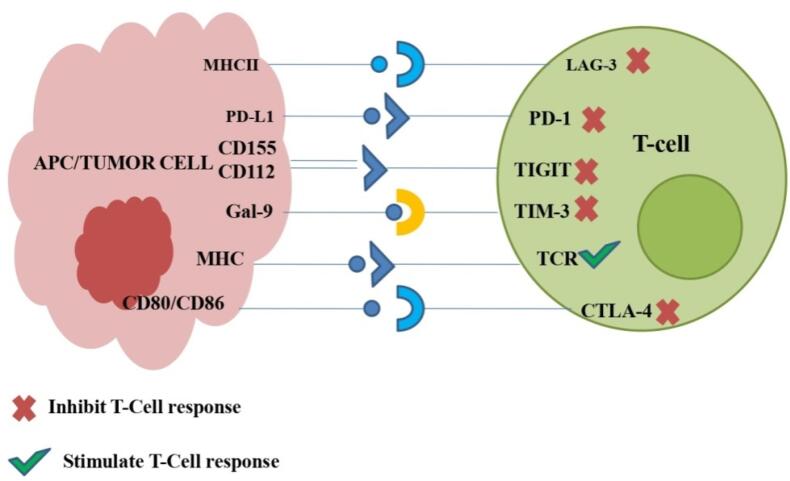
Lung cancer related Immunological checkpoints and their association with ligands: Following the remarkable success of PD-1/PD-L1 and CTLA-4, few other checkpoints have demonstrated the ability to control lung cancer. These recently identified checkpoints i.e., Lymphocyte activation gene-3 (LAG-3), T-cell immunoglobulin and mucin domain-3 (TIM-3), and T cell Ig and immunoreceptor tyrosine based inhibitory motif domain (TIGIT) are depicted in this figure. These identified checkpoints bind to specific ligands suppressing the response of T cell, while stimulatory signals are transmitted by TCR and MHC interaction

 Immunological checkpoint molecules are vital for regulating and sustaining the immune response. By suppressing the immunological response, these molecules located on surface of the immune cells stop autoimmunity. These immunological checkpoint molecules are produced by tumor cells in a TME to stop the immune system from developing tolerance to them. ICIs prevent the immunosuppressive process of tumor cells. Numerous monoclonal antibodies employed as ICIs attach to an antigen on a cancer cell and affect the subsequent signaling cascades and cell division.^15^ Figure 2 includes the identified ICIs and their ligands. The widely studied immune checkpoints are those that target Programmed death-1/ Programmed death- ligand 1 (PD-1/PD-L1) and Cytotoxic T-cell lymphocyte associated antigen (CTLA-4) pathway.^16^ The cancer cells exploit these pathways to promote immune suppression and proliferate unchecked.^17^ Cells like B-, T- and myeloid express the immune checkpoint regulator PD-1 on their surface, whereas tumor cells produce PD-L1 along with PD-L2, which binds to the PD-1 receptor. Binding of PD-1 to PD-L1 prevents CD8 ^+^ T-cells to identify cancer cells and thereby, avoid immune response. Further, this interaction suppresses B lymphocyte’s growth and its development to specialized cell types along with its ability to secrete Immunoglobulin (Ig) eventually diminish the immune response of activated cells. It inhibits T lymphocytes expansion and produces cytokines like IL-2 and IFN-γ.^18^ Thus, anti-PD-1/ PD-L1 drugs studied against various malignancies are now undergoing clinical trials to treat lung cancer.^19^ CTLA-4, an immunological checkpoint and a homolog of T-cell CD28, is displayed as a cell membrane receptor on activated CD8 + and CD4 + T-cells. The interaction of CD28 to B7 leads to T-cell multiplication and differentiation by producing cytokine like IL-2.^17^ The stimulatory signal of the binding of CD28-B7 and Major histocompatibility complex (MHC)- T cell receptor (TCR) can be countered by inhibitory signals generated due to the association of CTLA-4 to B7 instead of producing stimulatory activity.^20^ T-cell proliferation and activation can be promoted by blocking CTLA-4. ^21,22^

###  ICIs for lung cancer treatment

 In this section, we have covered the US Food and Drug administration (FDA) approved ICIs and other clinical trials meant for the treatment of lung cancer i.e., NSCLC and SCLC. Multiple trials involving ICIs have demonstrated encouraging outcomes even in late-stages of lung cancer improving survival either alone or in combination with chemotherapy. Table 1 presents the clinical trials of immune check point inhibitors for lung cancer along with their outcomes and current status.

**Table 1:** Clinical trials of immune check point inhibitors for lung cancer^23^

**Immune checkpoint inhibitors**	**Condition**	**Clinical trial ID**	**Phase**	**Enrollment**	**Start date**	**End date**	**Sponsor**	**Outcome/Status**
**Anti PD-1/PD-L1**								
Nivolumab (BMS-936558)	Previously treated Metastatic non-squamous NSCLC	NCT01673867	III	792	2/11/2012	17/12/2021	Bristol-Myers Squibb	After platinum-based chemotherapy approved by FDA in 2015
Nivolumab (BMS-936558)	Previously treated Metastatic non-squamous NSCLC	NCT01642004	III	352	16/10/2012	16/08/2021	Bristol-Myers Squibb	
Nivolumab monotherapy/ Nivolumab + Ipilimumab	ES-SCLC	NCT01928394	I/II	1163	24/10/2013	30/04/2023	Bristol-Myers Squibb	Active and not recruiting
Nivolumab/ chemotherapy	Previously treated Relapsed SCLC	NCT02481830	III	803	14/09/2015	22/08/2022	Bristol-Myers Squibb	Not effective
Nivolumab (Opdivo)/Nivolumab + Ipilimumab (Yervoy)/placebo as maintenance therapy	ES-SCLC after first-line chemotherapy	NCT02538666	III	1212	13/10/2015	11/11/2021	Bristol-Myers Squibb	Not effective
Nivolumab/Cisplatin/ Carboplatin with etoposide	ES-SCLC	NCT03382561	II	160	2/05/2018	9/06/2023	National Cancer Institute (NCI)	Active and not recruiting
Pembrolizumab (MK-3475) KEYTRUDA^®^/ docetaxel	Previously treated NSCLC	NCT01905657	II/III	1034	9/08/2013	30/09/2020	Merck Sharp & Dohme LLC	Pembrolizumab monotherapy for NSCLC patients approved by FDA in 2016
Pembrolizumab (MK-3475) KEYTRUDA^®^	Advanced or metastatic NSCLC	NCT01295827	I	1260	4/03/2011	11/12/2018	Merck Sharp & Dohme LLC	Dose of 2 mg/kg in previously treated patients might be effective
Pembrolizumab/ platinum-based chemotherapy	Untreated stage IV NSCLC	NCT02142738	III	305	25/08/2014	27/05/2021	Merck Sharp & Dohme LLC	Pembrolizumab monotherapy for NSCLC patients with high PD-L1 expression approved by FDA in 2016 as first-line treatment
Pembrolizumab + chemotherapy/immunotherapy	Unresectable or metastatic NSCLC	NCT02039674	I/II	267	21/02/2014	18/10/2021	Merck Sharp & Dohme LLC	Treatment of NSCLC irrespective of PD-L1 expression approved by FDA in 2017
Pembrolizumab (MK-3475)	Already treated SCLC	NCT02628067	II	1609	18/12/2015	18/06/2026	Merck Sharp & Dohme LLC	As third line treatment for SCLC patients approved by FDA in 2019
Atezolizumab (Tecentriq)	Locally advanced or metastatic NSCLC (PD-L1 Positive)	NCT01846416	II	138	30/05/2013	18/12/2017	Genentech, Inc.	1200 mg exposure is suggested
Atezolizumab	Locally advanced or metastatic NSCLC (PD-L1 Positive)	NCT02031458	II	667	22/01/2014	11/01/2019	Hoffmann-La Roche	1200 mg exposure is suggested
Atezolizumab (MPDL3280A)/ Docetaxel	Metastatic NSCLC patients with failed chemotherapy	NCT01903993	II	287	6/08/2013	6/09/2018	Hoffmann-La Roche	Well tolerated
Atezolizumab (Tecentriq)/Docetaxel	Metastatic NSCLC patients with failed chemotherapy	NCT02008227	III	1225	11/03/2014	9/01/2019	Hoffmann-La Roche	Approved by FDA after chemotherapy failure in NSCLC patients
Atezolizumab (Tecentriq)/chemotherapy consisting of a platinum agent	Stage IV NSCLC	NCT02409342	III	572	20/07/2015	8/03/2022	Hoffmann-La Roche	Significantly increased OS regardless of histologic type
Atezolizumab (Tecentriq) + Carboplatin + Paclitaxel/ Atezolizumab + Carboplatin + Nab-Paclitaxel/Carboplatin + Nab-Paclitaxel	Stage IV NSCLC	NCT02367794	III	1021	11/06/2015	17/02/2021	Hoffmann-La Roche	Atezolizumab as first and second line treatment for NSCLC approved by FDA in 2020
Atezolizumab + Carboplatin + Etoposide	ES-SCLC	NCT02763579	III	403	7/06/2016	8/07/2022	Hoffmann-La Roche	Improved OS
Durvalumab (MEDI4736)	Lung cancer	NCT01693562	I/II	1022	5/09/2012	28/02/2020	MedImmune LLC	Suggested further investigation
Durvalumab (MEDI4736)	Stage IIIB-IV NSCLC	NCT02087423	II	446	25/02/2014	30/06/2023	AstraZeneca	Active and not recruiting
Durvalumab/Docetaxel as second line treatment	Stage IV NSCLC	NCT02766335	II/III	116	July, 2014	September, 2020	Southwest Oncology Group	Increased PFS
Durvalumab (MEDI4736)	Stage III NSCLC	NCT02125461	III	713	7/05/2014	30/12/2022	AstraZeneca	As sequential therapy approved by FDA in 2018
Durvalumab + Tremelimumab + platinum based chemotherapy	Untreated ES-SCLC	NCT03043872	III	987	27/03/2017	30/12/2022	AstraZeneca	Active and not recruiting
Avelumab/Docetaxel	PD-L1 positive NSCLC after platinum based doublet failure	NCT02395172	III	792	24/03/2015	3/12/2019	EMD Serono Research & Development Institute, Inc.	Experienced prolonged response and clinical benefit
Cemiplimab/platinum based chemotherapy as first-line treatment	PD-L1 positive NSCLC	NCT03088540	III	712	29/05/2017	30/04/2024	Regeneron Pharmaceuticals	Treatment of advanced NSCLC by FDA in 2020.
**Anti-CTLA-4**								
Ipilimumab + Nivolumab	Recurrent or Metastatic NSCLC	NCT03527251	I	10	15/05/2018	31/12/2019	Sun Yat-sen University	Unknown
Ipilimumab (Yervoy) + Nivolumab (Opdivo) as first-line treatment	Stage IV or Recurrent NSCLC	NCT02477826	III	2748	5/08/2015	30/08/2024	Bristol-Myers Squibb	Combination in patients expressing high PD-L1 approved by FDA
Ipilimumab + Nivolumab	SCLC	NCT01928394	I/II	1163	24/10/2013	30/04/2023	Bristol-Myers Squibb	In previously treated SCLC patients approved by FDA in 2018

####  ICIs Targeting PD-1

**(i) Nivolumab:** Nivolumab was the first antibody against immune checkpoint PD-1 to undergo clinical studies for NSCLC.^24^ Following the success of CHECKMATE 057 and CHECKMATE 017, the FDA authorised nivolumab in 2015 for NSCLC treatment following platinum-derived chemotherapy. The third phase international trial called CHECKMATE 057 (NCT01673867) involved non-squamous NSCLC patients whose disease developed while or subsequently after platinum-based combination chemotherapy. These patients were grouped and received either nivolumab or docetaxel. The group receiving nivolumab exhibited a more favorable safety profile and prolonged the OS compared to the group receiving docetaxel. In a phase Ⅲ experiment called CHECKMATE 017 (NCT01642004) the safety and effectiveness of nivolumab and docetaxel were compared.^25^ However, CHECKMATE 032 (NCT01928394) and CHECKMATE 331 (NCT02481830), an early-phase (I/II) and phase III trial conducted on SCLC patients did not meet the expected endpoint and failed to prolong the OS. In another trial, CHECKMATE 451 (NCT02538666), platinum-derived chemotherapy treated SCLC patients underwent treatment with nivolumab alone, nivolumab + ipilimumab, and placebo as maintenance therapy. The trial did not reach the intended outcome and failed to prolong the OS. Furthermore, EA5161/NCT03382561, a second phase randomized study with nivolumab combined with etoposide (a chemotherapeutic agent) as first-line treatment for the patients of ES-SCLC. This study overall concluded that nivolumab with platinum and etoposide prolonged OS and PFS.^26^

**(ii) Pembrolizumab:** A humanised monoclonal IgG4-κ antibody called pembrolizumab targets the immunological checkpoint PD-1. In 2015, the KEYNOTE-010 (NCT01905657) trial was conducted on NSCLC patients with disease development after platinum-derived chemotherapy. In this study, the OS of pembrolizumab treated patients significantly improved in comparison to patients treated with docetaxel. Therefore, treatment with pembrolizumab alone for advanced NSCLC patients with disease progression was approved by the US FDA post-diagnosis in 2016. A phase I study, known as KEYNOTE-001 (NCT01295827), checked anti-cancer effectiveness and safety of pembrolizumab in NSCLC patients both treated and untreated.^27^ After the convincing results of KEYNOTE-001, another phase Ⅲ trial KEYNOTE-024 (NCT02142738) was conducted where high PD-L1 expressing untreated NSCLC patients were given pembrolizumab versus platinum-based chemotherapy. The outcome of KEYNOTE-024 led to approval for the use of pembrolizumab alone as a primary therapy against high PD-1 expressing NSCLC patients in 2016 by FDA.^28,29^ However, the major endpoint of KEYNOTE-024 was PFS. To study the effect of pembrolizumab monotherapy in advanced NSCLC with OS as the primary endpoint, KEYNOTE-042 was conducted and was found to be successful in 2018.^30^ The KEYNOTE-021 (NCT02039674), an early-phase (I/II) trial studied combined pembrolizumab and chemotherapy. In this study, the study group receiving pembrolizumab plus platinum-based chemotherapy showed significantly higher PFS.^31^ In 2017, irrespective of PD-1 expression pembrolizumab was approved by FDA for NSCLC treatment.^29^ A phase III study called KEYNOTE-189 compared effectiveness of pembrolizumab plus platinum-derived chemotherapy versus placebo plus platinum-derived chemotherapy in patients with untreated metastatic non-squamous NSCLC. Independent of PD-L1 expression, pembrolizumab + platinum-derived chemotherapy prolonged the OS and PFS. Overall KEYNOTE-021 and KEYNOTE-189 trial results justified pembrolizumab for NSCLC patients as primary treatment option. A phase III trial, KEYNOTE-407, conducted on squamous NSCLC patients verified the use of pembrolizumab + chemotherapy as the primary treatment.^32^ also confirmed OS and PFS improvements with manageable toxicity consistent with previous reports.^33^

 Pembrolizumab has been used in a total of seven clinical trials to treat SCLC. A phase 1b trial, KEYNOTE-028 tested the effectiveness of pembrolizumab in patients with SCLC expressing high PD-L1. KEYNOTE-158 (NCT02628067), a phase II trial later demonstrated pembrolizumab’s effective anti-tumor efficacy in late-stage SCLC patients and PD-L1 positive tumors. Both trials were conducted with already treated SCLC patients.^34^ According to the sustained response observed in the trials, the US FDA in 2019 approved pembrolizumab to be used as the tertiary treatment of ES-SCLC patients whose disease progressed following platinum-derived chemotherapy. A third phase trial, KEYNOTE-604, explored the use of pembrolizumab and platinum (Carboplatin/Cisplatin) + etoposide in untreated SCLC patients. However, the outcome of KEYNOTE-604 showed that pembrolizumab is unlikely to gain approval as a primary treatment option in SCLC patients.^35^ The efficiency of pembrolizumab has also been studied in ES-SCLC patients after platinum and etoposide-based therapy as maintenance therapy. However, the studies failed to meet their primary endpoints.^36^

**(iii) Atezolizumab:** Atezolizumab is a humanised monoclonal IgG1 anti-PD-L1antibody. The anti-tumor efficiency of non-comparative atezolizumab monotherapy of FIR (NCT01846416) along with BIRCH (NCT02031458) were assessed through phase II trials.^37^ POPLAR (NCT01903993), a phase II trial with platinum-derived chemotherapy treatment was conducted in patients with late-stage NSCLC. The trial compared the activity of atezolizumab with docetaxel and found that patients receiving atezolizumab significantly improved OS in comparison to patients receiving docetaxel.^38^ Regardless of expression of PD-L1, OAK (NCT02008227), another late-phase (III) trial, was carried out among NSCLC patients who had undergone a minimum of one platinum-derived treatment. The patients receiving atezolizumab showed higher OS compared to patients receiving docetaxel.^39^ Atezolizumab is the first anti-PD-L1 antibody that FDA approved for NSCLC patients whose disease progressed after receiving platinum-derived chemotherapy.^40^ The IMpower project was carried out to evaluate the efficiency of atezolizumab monotherapy or in combination for NSCLC patients expressing PD-L1 as initial treatment. IMpower 110 (NCT02409342) and IMpower 111 (NCT02409355), phase III trials assessed the activity of atezolizumab monotherapy versus platinum-derived chemotherapy in PD-L1 positive advanced NSCLC patients.^41^ IMpower 130, 131, and 132 are phase III trials to compare atezolizumab combined with chemotherapy and standardized platinum-based chemotherapy.^42^ IMpower 130 showed OS and PFS improvement due to atezolizumab + chemotherapy and supported the benefit of the combination for NSCLC as first-line treatment.^43^ Additionally, the outcomes of IMpower 131 (NCT02367794) aligned with earlier studies that backed the application of atezolizumab as a primary and subsequent treatment option for NSCLC.^44^ Atezolizumab was given FDA approval in 2020 as adjuvant treatment in advanced high PD-L1 expressing NSCLC patients with post-resection and platinum-based chemotherapy.^45^

 In several countries like Canada, Japan, China, the US and the EU, atezolizumab was permitted as a frontline treatment for ES-SCLC treatment along with Carboplatin and etoposide. Atezolizumab’s approval was predicated on IMpower 133’s primary results.^46^ A third phase trial named IMpower 133 (NCT02763579) examined the effectiveness and safety of atezolizumab plus carboplatin and etoposide as primary therapy for patients with SCLC.^47^

**(iv) Durvalumab:** Durvalumab is a human IgG1 κ monoclonal antibody specifically designed to target PD-L1.^48^ NCT01693562 was the first study on humans to test durvalumab in advanced lung cancer patients, initially including all patients but later focusing only on those with PD-L1 expression. Safety and tolerability were the major endpoints of the study. In ATLANTIC (NCT02087423), a phase II trial was conducted among late-stage NSCLC patients as first-line and second-line treatment. S1400A Lung-Map umbrella (NCT02766335), a phase II study evaluated durvalumab monotherapy versus docetaxel as second-line treatment. Durvalumab was approved by FDA in 2018 as a second-line treatment for NSCLC patients after chemoradiation.^49^ Durvalumab obtained FDA approval due to the compelling outcomes of the PACIFIC trial (NCT02125461), a phase III study involving non-resectable stage III NSCLC patients with the disease stable after 12 months of platinum-derived concurrent chemoradiation. This treatment demonstrated significant OS and PFS improvement.^50^ A phase II trial, SAPHIR02 Lung trial (NCT02117167), is ongoing to assess the activity of durvalumab after chemotherapy as maintenance treatment.

 In a phase III trial called CASPIAN (NCT03043872), durvalumab combined with etoposide and carboplatin/cisplatin was assessed for SCLC patients. Durvalumab has significantly improved OS rates when used in conjunction with platinum etoposide chemotherapy as the frontline-treatment option for ES-SCLC. Durvalumab is approved in countries such as the US, China, Japan, the EU, and many others. The clinical practice guidelines of National comprehensive cancer network (NCCN) along with the European Society for medical oncology (ESMO) includes recommendations for using durvalumab combined with etoposide and Carboplatin/cisplatin for ES-SCLC patients.^51^

**(v) Avelumab:** Avelumab, a human monoclonal IgG1 antibody targeted against PD-L1 was developed by Merck, Darmstadt, Germany and currently is in Phase II for SCLC. As per the report by Global Data, 26% phase transition success rate of avelumab for SCLC indicated the likelihood of approval and progressing into Phase III (2023, July 1).^52^ The clinical trials of avelumab monotherapy or in combination were done as a part of the international JAVELIN clinical study program.^53^ JAVELIN Lung 200 (NCT02395172), a phase III trial was performed after avelumab showed clinical activity in late-stage NSCLC patients as second-line treatment. However, the trial did not significantly improve the OS compared to patients administered with docetaxel.^54^

**(vi) Cemiplimab:** Cemiplimab, a monoclonal IgG4 antibody targeting PD-L1 approved to treat metastatic cutaneous squamous cell carcinoma.^55^ The effectiveness of cemiplimab monotherapy and platinum-derived chemotherapy as a primary treatment for late-stage NSCLC expressing PD-L1 was compared in the EMPOWER-Lung 1 (NCT03088540) phase III trial. Cemiplimab showed clinically substantial extension of OS and PFS.^56^ FDA approved the monotherapy of cemiplimab to treat advanced-stage NSCLC in 2020 depending on the outcomes of EMPOWER-Lung 1 trial.

####  ICIs targeting CTLA-4

**(i) Ipilimumab:** Ipilimumab is an only anti-CTLA-4 monoclonal antibody (fully human IgG1) approved by the US FDA for cancer treatment. Ipilimumab as monotherapy has not yet shown any efficacy in NSCLC. However, ipilimumab combined with chemotherapy and PD-L1 inhibitors has demonstrated encouraging outcomes in both preclinical and clinical research.^57^ A phase I trial called CheckMate-012 (NCT03527251) assessed the effectiveness of ipilimumab plus nivolumab in untreated NSCLC patients. The phase II trial, CheckMate 568 confirmed the high response rates in the case of ipilimumab + nivolumab. CheckMate 227, phase III clinical trial investigated treatment-related safety and activity of ipilimumab plus nivolumab as frontline therapy in NSCLC patients.^58^ Based on its results, the US FDA authorized the use of ipilimumab and nivolumab as a first-line therapy for NSCLC patients expressing high metastatic PD-L1.^59^

 A phase III trial called CheckMate 451 evaluated the efficacy and safety of ipilimumab + nivolumab in SCLC patients. However, the study’s main goal of extended OS was not met.^60^ A phase I/II experiment called CheckMate 032 evaluated the activity of ipilimumab + nivolumab in patients with SCLC who had undergone previous treatment. Patients treated with nivolumab + ipilimumab showed superior clinical benefits in comparison to the monotherapy in the high tumor mutation burden group. Based on its results, in 2018, the use of ipilimumab plus nivolumab was authorised by FDA in previously treated SCLC patients.^61^

**(ii) Tremelimumab:** Tremelimumab (CP-675, 206) is a human IgG2 anti-CTLA4 monoclonal antibody. Tremelimumab is different from ipilimumab with respect to having different ADCC and CDC activities.^62^ The US FDA has approved tremelimumab (Imjudo, AstraZeneca Pharmaceuticals) + durvalumab (Imfinzi, AstraZeneca Pharmaceuticals) along with platinum containing chemotherapy for metastatic NSCLC adult patients (2022, November 10).^63^

## Lung cancer vaccines

 Activating the adaptive anti-tumor immune response is the primary goal of cancer vaccine therapy. The majority of the cancer vaccines concentrate on targeting specifically the known or unknown tumor-associated antigens (TAAs). The list of vaccines that have been studied in NSCLC and SCLC is shown in Table 2.

**Table 2:** Clinical trials of vaccines for lung cancer^23^

**Target**	**Trade name**	**Clinical Trial ID**	**Indication**	**Status**	**Phase**	**Participants**	**Start date**	**End date**	**Sponsor**
Belagenpumatucel -L	Lucanix	NCT01058785	Stage II-IV NSCLC	Completed	II	75	March, 2003	July, 2010	NoraRx Corporation
GM-CSF producing irradiated autologous vaccine	GVAX	NCT00074295	Stage IIIB and IV lung cancer	Terminated due to lack of availability of vaccine	II	19	March, 2004	July, 2012	Southwest Oncology Group, National Cancer Institute (NCI)
**Peptide/protein vaccines**									
MAGE-A3	Imvamune, Imvanex	NCT04908111	NSCLC	Recruiting	II	86	8/12/2021	May, 2025	Cancer Research, UK
MAGE-A3		NCT02879760	Previously treated Metastatic NSCLC	Completed	I/II	16	8/03/2017	24/05/2020	Turnstone Biologics, corp
MUC1	Tecemotide (L-BLP25)	NCT00409188	Stage III NSCLC	Completed	III	1513	January, 2007	April, 2015	EMD, Serono
MUC1	Tecemotide (L-BLP25)	NCT00960115	Stage III unresectable NSCLC	Completed	I/II	178	December, 2008	June, 2015	Merck KGaA, Darmstadt, Germany
EGF	CIMAvax-EGF	NCT04298606	NSCLC	Recruiting	Early Phase I	60	22/11/2021	19/04/2023	Roswell Park Cancer Institute
MUC1	Stimuvax	NCT01720836	NSCLC	Recruiting	I/II	30	November, 2012	September, 2029	Olivera Finn, University of Pittsburgh
CEA	Ad5 [E1-, E2b-]**-**CEA(6D) or ETBX-011	NCT01147965	Metastatic CEA positive lung cancer	Completed	I/II	35	June, 2010	March, 2013	Etubics Corporation
IDO		NCT01219348	Locally advanced or metastatic NSCLC	Completed	I	14	June, 2010	August, 2012	Inge Marie Svane, Herlev Hospital
**Dendritic cell vaccines**									
**CEA** RNA-pulsed DC cancer vaccine		NCT00004604	Lung cancer	Completed	I	24	February, 1997	July, 2002	Duke University
Personalized peptides loaded DC	PEP-DC vaccine	NCT05195619	Metastatic or advanced NSCLC	Recruiting	I	16	10/12/2021	September, 2024	Centre Hospitalier Universitaire Vaudois
Personalized neoantigen-primed dendritic cell vaccines		NCT03871205	Lung cancer	Unknown	I	30	1/04/2019	30/12/2020	Shenzhen People's Hospital
Dendritic cells transduced with an adenoviral vector containing the p53 Gene		NCT00617409	ES-SCLC	Completed	II	69	2/10/2007	31/01/2019	H. Lee Moffitt Cancer Center and Research Institute
Adenovirus-transfected autologous DC vaccine plus cytokine-induced killer (CIK) cells		NCT02688673	ES-SCLC	Active, not recruiting	I/II	30	August, 2014	November, 2016	Affiliated Hospital to Academy of Military Medical Sciences

###  Cell-based vaccines

 Cell-based vaccines utilize killed or inactivated tumor cells. The tumor derived from patients (autologous) or established cancer cell lines of related tumour types (allogeneic) can be used to harvest these tumour cells. GVAX is a genetically engineered irradiated autologous or allogeneic to secrete Granulocyte-macrophage colony stimulating factor (GM-CSF).^64^ GVAX’s first round of clinical study included patients with stage IV NSCLC, demonstrated favorable safety and tolerability profiles. The results demonstrated that after vaccination the patients secreting GM-CSF showed longer survival. Another trial using unmodified cancer cells co-cultured with allogeneic cell line K562 (a human erythroleukemia cell line) genetically engineered to produce elevated amount of GM-CSF. However, this approach did not demonstrate survival benefits in the patients. Despite the encouraging outcomes from the early trials of GVAX in phase I/II, the results of VITAL trials which compared GVAX and chemotherapy did not encourage conducting of phase III trials.^65^

 Four NSCLC cell lines were used to create the allogeneic tumor cell vaccine called Belagenpumatucel-L. All the transfected cell lines exhibited knockdown of transforming growth factor β2 (TGF-β2) gene. The vaccine promoted the suppression of NSCLC cells by acting of cytotoxic T lymphocytes (CTL) response and enhancing the immune response suggesting phase Ⅲ trial.^66^ The effectivity of Belagenpumatucel-L as a maintenance therapy in stage III and IV NSCLC patients was carried out through a phase III trial called STOP. Although it did not considerably enhance OS, more research was necessary.^67^

###  Peptide or protein vaccines

####  CIMAvax-EGF

 CIMAvax-EGF is the first lung cancer vaccine registration. The identification of epidermal growth factor receptor (EGFR) overexpression in lung cancer led to the creation of CIMAvax-EGF. Through a variety of intracellular pathways, blocking the EGF–EGFR connection that results in the loss of a signalling network is essential for inhibiting apoptosis, tumor growth, cellular differentiation and transformation, cell migration and invasion.^68^ In the last two decades, more than 10 clinical trials were conducted on the use of CIMAvax-EGF after registration. It has shown a major effect on survival mainly in patients completing the fourth induction dose and in patients selected based on high EGF concentration. Clinical trials to combine CIMAvax-EGF and anti-PD-1/PD-L1 antibodies are also conducted.^69^

####  Melanoma-associated antigen-A3 (MAGE-A3) 

 The MAGE-A3 vaccine targets an antigen expressed by cancer cells in melanoma and lung cancer showing the highest expression percentage. In combination with an appropriate adjuvant, MAGE-A3 has been clinically tested in NSCLC patients.^70^ The phase III trial MAGRIT in surgically resected NSCLC patients that are MAGE-A3 positive did not show an increase in PFS compared to the placebo and therefore further research has been stopped.^40^

####  Liposomal MUC1 vaccine BLP25 (L-BLP25)

 The peptide vaccine, L-BLP25 that targets Mucinous transmembrane glycoprotein, MUC1 tumor-associated antigen that is an integral membrane protein with extensive glycosylation. Overexpression of MUC1 by cancer cells confers survival benefits under stress conditions. The BLP25 is a lipopeptide containing 25 amino acid sequences that provide MUC1 specificity.^71^ After the encouraging results of the phase II trial (START), a phase III trial and INSPIRE were conducted in NSCLC patients. Nevertheless, the studies could not meet the endpoints of prolonged OS and PFS.^72,73^

####  Indoleamine 2, 3-dioxygenase (IDO)

 IDO is an intracellular enzyme which catalyzes a rate-limiting step involved in tryptophan metabolism. Tumor cells are found to upregulate IDO which leads to tryptophan depletion suppressing T-cell function and survival. This suggests that IDO is a valued target in cancer. A first phase vaccination trial in 2010 showed that the vaccine-induced PD-L1 regulation in tumor cells and immune cells increasing the susceptibility towards PD-1/PD-L1 immunotherapy. So, IDO-derived peptide vaccine combined with PD-1 antibody potentially increases clinical benefits.^74^

 Another peptide vaccine IDM-2101 was developed against five overexpressed tumor antigens {i.e., P^53^ HER2/ *neu*, Carcinoembryonic antigen (CEA), MAGE-2 and -3} found in NSCLC. The second phase clinical study for IDM-2101 was conducted in HLA-A2 positive NSCLC patients. The results showed that the vaccine induced a durable immune response, however further safety and efficacy of the vaccine were suggested.^75^

###  Dendritic cell vaccines

 In recent years, the application of patient derived dendritic cells (DCs) co-cultured with cytokine-stimulated killer cells for lung cancer has undergone clinical trials. Additionally, the design of patient-specific neoantigen dendritic cell vaccines has emerged as a novel approach in this field. Tumor-specific antigens known as neoantigens are produced when the tumour genome undergoes non-synonymous somatic mutations. Neoantigens are specific to tumors, in contrast to TAAs, which can also be found in normal cells. This distinction makes neoantigens promising targets for drug development. Several neoantigen-loaded DC vaccines are also being clinically tested in lung cancer patients. DC vaccines combined with chemotherapy, radiotherapy, or other ICIs can lead to the success of DC-based vaccines.^76^

###  DNA vaccines

 The main concept of DNA vaccine is the introduction of potentially therapeutic TAAs into the host and the stimulation of the immune defenses in the host. The advancement of effective and site-specific delivery systems for DNA vaccines is very important for the vaccine to be effective. A modified bacterium, *Salmonella enterica *serovarTyphimurium live attenuated strain, SL3261 possesses a mutant form of the aloA gene related to aromatic amino acid. Oral DNA vaccines containing SL3261 that target the vascular endothelial growth factor receptor-3 (VEGFR-3) extracellular domains have shown efficacy in treating melanoma, colon cancer, lung cancer and breast cancer. According to the findings of the study, tumor lymphatic microvessels may be destroyed by oral VEGFR-3-based DNA vaccines that trigger an immune reaction against endothelial cells.^77^

###  Viral vector-based vaccines

 The phase II/III trial of NCT00415818, investigated the efficacy of vaccine TG4010-based viral vector when combined with chemotherapy and were compared to chemotherapy alone in patients with advanced NSCLC. The viral vector is the modified vaccinia virus Ankara (MVA), which encodes for human MUC1 and IL-2. The trial findings showed that the TG4010 was well tolerated and capable of achieving OS and PFS, the primary endpoints, when used in conjunction with first-line chemotherapy.^78,79^ Based on the results a phase IIB/III study (NCT01383148) was conducted. Another study (NCT03353675) with TG4010 vaccine with ICI immunotherapy (nivolumab) was conducted in late-stage NSCLC patients as frontline treatment option.

## Adoptive cell therapy

 Adoptive cell therapy is a promising method within immunotherapy, involving the cultivation of tumor-reactive lymphocytes, primarily CTLs, from patients in an ex-vivo environment. These enhanced cells are subsequently reintroduced into the patient to target and combat tumors effectively. Modified TCR therapies involve enhancing T-cell specificity by expressing particular TCRs that facilitate the antigen-recognition process.^80^ Among these, chimeric antigen receptor-T (CAR-T) cell therapy is approved by the US FDA for the treatment of cancer. In case of lung cancer, various targets like HER2, EGFR, MAGE-A1, MUC1, mesothelin (MSLN), CEA, inactive Tyrosine-protein kinase transmembrane receptor ROR1, PD-L1, B7-H3 and Chemokine receptor CXC4 have been identified and clinical studies have already been performed. Several other identified TAAs for lung cancer, like the folate receptors α and β, tyrosine kinase receptor EphA2, phosphatidylinositol proteoglycan 3 GPC3, CD44v6, Lewis-Y antigen, IL-13Rα2, L1 cell adhesion molecule (L1CAM) and disialoganglioside GD2, are yet to be tested.^81,82^

 EGFR, or HER1, is an integral membrane glycoprotein that is part of the ErbB receptor protein-tyrosine kinase family. More than 60 percent of EGFR mutations are associated with tumor growth and metastasis in NSCLC. CAR-T cells against EGFR in clinical studies have shown cytotoxic activity and high cytokines levels such as interleukin -2, -4, -10, Tumor Necrosis Factor-α, and Interferon-γ. Anti-EGFR CAR-T therapy shows promise for treating EGFR-positive lung carcinoma, but additional clinical validation is necessary. MUC1 is another transmembrane glycoprotein which is upregulated in NSCLC. The MUC1 CAR-T cells have shown encouraging results in preclinical studies. MSLN is a glycosylphosphatidylinositol-anchored protein found on the cell surface, which many tumors express at high levels, particularly lung cancer. Currently, several early-stage ongoing clinical trials are underway to evaluate the efficacy of MSLN CAR-T cell therapy.^23,82,83^ Many other TAAs have been identified and clinically tested, which can be accessed through ClinicalTrials.gov as presented in Table 3.

**Table 3:** Clinical trials of CAR-T cell therapy on TAAs of lung cancer^23^

**TAAs**	**Clinical trial ID**	**Phase**	**Indication**	**Estimated enrollment**	**Recruitment status**	**Study start date**	**Estimated study completion date**	**Sponsor**
EGFR	NCT05060796	Early Phase I	Advanced NSCLC	11	Recruiting	1/09/2019	1/11/2034	Second affiliated hospital of Guangzhou medical university, China
EGFR	NCT04153799	I	Advanced NSCLC	11	Recruiting	1/11/2019	1/12/2022	Sun Yat-sen University, China
EGFR	NCT01869166	I/II	Advanced EGFR positive NSCLC	60	Unknown	1/05/2013	1/11/2017	Chinese PLA General Hospital
MUC1	NCT02587689	I/II	MUC1 positive NSCLC	20	Unknown	1/10/2015	1/10/2018	PesonGen Bio Therapeutics (Suzhou) Co., Ltd.
MUC1	NCT03525782	I/II	MUC1 positive NSCLC	60	Unknown	1/02/2018	31/02/2022	First affiliated hospital of Guangdong pharmaceutical university
MSLN	NCT01583686	I/II	Metastatic/unresectable MSLC positive NSCLC	15	Terminated	4/05/2012	17/11/2018	National Cancer Institute
MSLN	NCT03054298	I	Metastatic/recurrent lung adenocarcinoma	27	Recruiting	6/04/2017	1/03/2025	University of Pennsylvania
MSLN	NCT02414269	I/II	Malignant NSCLC	113	Active, not recruiting	1/05/2015	30/04/2024	Memorial Sloan Kettering cancer center
ROR1	NCT02706392	I	Metastatic/unresectable NSCLC	21	Terminated due to slow accrua	16/03/2016	28/09/2021	Fred Hutchin Cancer Research Center, USA
CEA	NCT04348643	I/II	Metastatic or recurrent CEA positive lung cancer	40	Recruiting	20/02/2020	30/04/2023	Chongqing Precision Biotech Co., Ltd., China
CEA	NCT02349724	I	Relapsed or refractory CEA positive lung cancer	75	Unknown	1/12/2014	1/12/2019	Southwest Hospital, China
HER2	NCT03740256	I	HER2 positive lung cancer	45	Recruiting	14/12/2020	30/12/2038	Baylor College of Medicine, USA
HER2	NCT0193584	I/II	Advanced chemotherapy refractory HER2 positive lung cancer	10	Unknown	1/09/2013	1/09/2017	Chinese PLA General Hospital, China
HER2	NCT04660929	I	HER2 positive metastatic/recurrent lung cancer	18	Recruiting	2/02/2021	1/02/2023	Carisma Therapeutic Inc., USA
HER2	NCT02713984	I/II	Relapsed and refractory HER2 positive lung cancer	0	Withdrawn due to safety consideration	1/03/2016	1/07/2019	Zhi Yang, Southwest Hospital, China
HER2	NCT01935843	I/II	Chemotherapy refractory HER2 positive NSCLC	10	Unknown	1/09/2013	1/09/2017	Chinese PLA General Hospital, China
PD-L1	NCT02862028	I/II	Advanced EGFR/HER2/HER4/IGFR1 positive lung cancer	20	Unknown	August, 2016	July, 2018	Shanghai International Medical Center, China

## Limitations and challenges of current lung immunotherapy

 Cancer treatment with immunotherapy has significantly improved the longevity of patients in a substantial proportion. Despite significant progress in precision therapies, late-stage lung cancer patients have not seen a notable reduction in mortality rates. One significant drawback is the high cost, which restricts both accessibility and sustainability in the healthcare sector.^84^ This emphasizes the need to develop strategies for cheaper and affordable immunotherapy treatments for a larger population. The therapeutic effectiveness of immunotherapy works by stimulating the immune response and also on addressing the intricate complexities of cancer biology. Tumor heterogeneity, characterized by the presence of various cell populations within a single tumor, poses a considerable challenge to the success of immunotherapy. The genetic profiling of lung cancer has revealed significant heterogeneity among patients. This research has successfully identified multiple oncogenes and tumor suppressor genes, including RET, ROS1, BRAF V600E, KRAS, TP53, c-MET, NTRK, and ERBB2 (HER2). This leads to variability in the patient’s response to immunotherapy.^85^ More than 20 immunotherapy regimens has been approved by the FDA for the treatment of lung cancer since the first approval of ICIs for this purpose in 2015. As immunotherapy becomes increasingly widespread in the treatment of lung cancer, the occurrence of immune-related adverse events (irAEs) has increased. The irAEscan be severe and also life-threatening. The common irAEs related to immunotherapy in lung cancer are skin rash, hepatitis, hypothyroidism, pneumonitis, diarrhea and colitis, etc. With the increase of irAEs, emerging treatment methods and advancements are contributing to better patient outcomes. Continuous monitoring of the irAEs and their symptoms are suggested for their management.^86^ Cancer patients are also found to be resistant to immunotherapies, which can be characterized into primary and acquired resistance. These resistances have been seen in the case of ICIs. Therefore, understanding the underlying mechanism of resistance to ICIs has become a major challenge in cancer immunotherapy. Resistance to previous therapies frequently arises from the tumor’s ability to adapt, allowing it to evade immune detection or from the establishment of an immunosuppressive TME. Current strategies should also prioritize addressing the challenges by specifically targeting immunosuppressive cells within the TME and reprogramming immune cells to maintain a sustained attack.

## Research gaps and future perspectives in lung cancer immunotherapy

 Current research should concentrate on comprehending the pathophysiology and developing new strategies to lessen the undesirable immune-related consequences linked to diverse immunotherapies. It is vital to create trustworthy research models to analyze the effectiveness and adverse effects of cancer immunotherapy. Although they are still in their early stages, technologies like humanised mice, *in vitro* co-culture systems, organoids and microfluidic-based organoids-on-a-chip models have enormous research promise. Many abnormalities in the signaling pathway of JAK/STAT, MAPK, and WNT/β-catenin are associated with resistance against ICIs. The cases of resistance are mostly found in patients with progressing cancer. The CTLA-4 and PD-1/PD-L1 immune checkpoints have provided a remarkable prognosis for lung cancer treatment however, but all the lung cancer patients are not benefited by ICIs. The unpredictable efficacy of the patients depends on tumor heterogeneity, immunosuppressive TME and lack of predictive biomarkers. Biomarker-driven immunotherapy serves as a fundamental element of this tailored approach. Apart from PD-1/PD-L1, other cutting-edge biomarkers like CRP, LDH, MMR, VEGF, GEF, Tumor-infiltrating lymphocytes (TIL), TMB, etc. need validation through adaptive clinical trials. To increase the potential of ICIs and to provide immunotherapeutic advantages, other immune checkpoints are also being investigated.^84^ Among these checkpoints are LAG-3, LAG-3, Human Endogenous Retrovirus- H Long Terminal Repeat-Associating Protein-1 (HHLA2), V-Domain Ig Suppressor of T cell activation (VISTA), TIM-3 and TIGIT. Gaining insight into biological mechanisms regulating immune checkpoints will aid in the development of effective combination therapies and help address risk of resistance.^16^ Cancer vaccines have exhibited very limited clinical benefit mainly due to the difficulty in locating specific target tumor antigens (neoantigens) that are distinctive and overexpressed by tumor cells in contrast to normal tissues. The implication of AI and machine learning in identifying neoantigens holds significant promise for advancing personalized cancer immunotherapy, particularly in lung cancer treatment, by enabling the development of highly targeted and patient-specific cancer vaccines. AI methods that can forecast MHC-Ⅰ/Ⅱ binding efficacy and immunogenicity include TSNAD, pVAC-Seq, MARIA, EDGE, INTEGRATE-neo, and others. AI algorithms can efficiently evaluate vast quantities of potential neoantigens, pinpointing the most promising candidates for vaccine development. By forecasting MHC binding affinity, AI aids in the selection of novel antigens that are most capable of stimulating a robust immune response against cancer cells.^87,88^

 Clinical trials have reported positive results with CAR-T cell therapy in lung cancer patients. It demonstrates stronger target-binding capability, along with a faster and longer-lasting therapeutic effect in lung cancer patients. However, it carries some side effects and associated risks.^89-91^ Future research on CAR-T cell therapy is suggested to focus on the improvement of tumor penetration of the therapy, better understanding of tumor resistance mechanism and development of better combined therapies using more rigorous and comprehensive bioinformatics approach.^81^ Another approach, TIL therapy have been introduced as an innovative immunotherapeutic method where TILs are extracted from the patient’s tumor and grown in culture with IL-2. IL-2 is found to activate tumor-killing activity. Studies have been conducted with TIL therapy in solid tumor including lung cancer. Further clinical trials with TIL therapy on lung cancer are expected to bring new edges to cancer immunotherapy.^85^

## Conclusion

 Several immunotherapies for lung cancer have been clinically tested and some of them are approved for lung cancer treatment worldwide. Although immunotherapy can lengthen survival in people with lung cancer, response rates are often poor. It is unknown which traits are responsible for the variation in efficacy and survival immunotherapy. Apart from patients’ response rate, the high cost has limited its usage. The incidences of irAEs and immunotherapy resistance need research attention. Future research must focus on treatments combining chemotherapy, vaccination, and ICIs, identification of new targets using several bioinformatics and AI tools to speed up. Addressing these challenges and research gaps will enable the wider use of immunotherapy for lung cancer treatment.

## Competing Interests

 There are no conflicts of interest.

## Ethical Approval

 Not Applicable.
